# MEK inhibition suppresses B regulatory cells and augments anti-tumor immunity

**DOI:** 10.1371/journal.pone.0224600

**Published:** 2019-10-31

**Authors:** Mark Yarchoan, Aditya A. Mohan, Lauren Dennison, Teena Vithayathil, Amanda Ruggieri, Gregory B. Lesinski, Todd D. Armstrong, Nilofer S. Azad, Elizabeth M. Jaffee

**Affiliations:** 1 Bloomberg–Kimmel Institute for Cancer Immunotherapy, Johns Hopkins University School of Medicine, Baltimore, MD, United States of America; 2 Department of Hematology and Medical Oncology, Winship Cancer Institute of Emory University, Atlanta, GA, United States of America; University of California, San Francisco, UNITED STATES

## Abstract

Mitogen-activated protein kinase (MAPK) kinase (MEK) is an integral component of the RAS pathway and a therapeutic target in RAS-driven cancers. Although tumor responses to MEK inhibition are rarely durable, MEK inhibitors have shown substantial activity and durable tumor regressions when combined with systemic immunotherapies in preclinical models of RAS-driven tumors. MEK inhibitors have been shown to potentiate anti-tumor T cell immunity, but little is known about the effects of MEK inhibition on other immune subsets, including B cells. We show here that treatment with a MEK inhibitor reduces B regulatory cells (Bregs) *in vitro*, and reduces the number of Bregs in tumor draining lymph nodes in a colorectal cancer model *in vivo*. MEK inhibition does not impede anti-tumor humoral immunity, and B cells contribute meaningfully to anti-tumor immunity in the context of MEK inhibitor therapy. Treatment with a MEK inhibitor is associated with improved T cell infiltration and an enhanced response to anti-PD1 immunotherapy. Together these data indicate that MEK inhibition may reduce Bregs while sparing anti-tumor B cell function, resulting in enhanced anti-tumor immunity.

## Introduction

The mitogen-activated protein kinase (MAPK) cascade is a critical pathway for cell proliferation and inhibition of apoptosis and is one of the most frequently dysregulated driver pathways in cancer [1]. Aberrant activation of the MAPK pathway resulting from activating mutations in *RAS* or *RAF* is observed in a wide number of human cancers including many melanomas, non-small cell lung cancers, colorectal cancers, and other tumor types. Mitogen/Extracellular signal regulated Kinase (MEK) is an intermediary component of the MAPK pathway. Although MEK itself is rarely mutated in human cancers, it is a downstream effector of mutant alleles of Rapidly Accelerated Fibrosarcoma (RAF) or RAS and therefore mediates constitutive activation of the MAPK pathway [2]. Multiple small-molecule inhibitors of MEK have been developed and have shown clinical activity in tumors with MAPK activation both alone and in combination with other targeted therapies [3–5]. However, due to the emergence of drug resistant clones, tumor responses to targeted inhibition of the MAPK pathway are rarely durable. By contrast, novel immune checkpoint inhibitors targeting programmed cell death protein 1 (PD-1) or its ligand, programmed death-ligand 1 (PD-L1), have the potential to transform short lived responses observed with targeted therapies into durable and clinically meaningful responses. Therefore, there is a significant clinical interest in combining MEK inhibition with immunotherapies [6,7].

MEK inhibitors have shown substantial efficacy when combined with PD-1 immunotherapy in a murine model of colon cancer and melanoma [8][9]. However, the mechanisms underlying the improved anti-tumor immune response with MEK inhibitors are complex. Notably, MEK signaling is a key pathway involved in both tumor cell survival and lymphocyte response to antigen stimulation. In support of this notion, MEK inhibition can block the priming of naive T cells *in vitro* and in lymph nodes *in vivo*. Unexpectedly, however, pharmacologic MEK inhibition has been shown to increase T-cell accumulation within tumors in both preclinical models and humans [9–11]. Several explanations have been proposed for why MEK inhibition may improve T cell responses in models of established tumors. MEK inhibition may rescue MAPK-mediated immune suppression by increasing the expression of major histocompatibility complex class 1 (MHC-I) on tumor cells, resulting in increased tumor antigen recognition by T cells [9,11]. MEK inhibition may also potentiate anti-tumor T cell immunity by impairing TCR-driven apoptosis [9]. However, the effect of MEK inhibition on other immune subsets that can modulate the anti-tumor immune response is not known.

An emerging body of evidence has recently recognized a role for B cells in modulating the immune response in cancer and other diseases for which T cells are recognized as the primary downstream effectors. Similar to regulatory T cells, regulatory B cells (Bregs) are a heterogeneous subset of B cells with distinct cell surface markers that can suppress effector T cell function and promote immune tolerance. Described subsets of Bregs in mice include B10 cells (CD19^+^CD5^+^CD1d^hi^), TIM1+ B cells (CD19^+^TIM1^+^), and T2-MZP cells (CD19^+^CD21^hi^CD23^hi^CD24^hi^)[12]. T2-MZP, in particular, have been implicated in tumor progression because they can accumulate in tumor draining lymph nodes, where they may facilitate an immunosuppressive environment and attenuate anti-tumor immune responses [13]. The MAPK pathway is downstream of B cell antigen receptor (BCR) signaling and is necessary for a subset of B cell responses to antigen [14]. However, despite the increasing evaluation of MEK inhibitors in the treatment of many cancer types, little is known about the effects of MEK inhibitors on B cell function. Here, we propose that the reprogramming of B cells may be a novel immunomodulatory mechanism through which MEK inhibition can enhance anti-tumor immunity. We show that MEK inhibition reduces Bregs *in vitro* and *in vivo* while preserving anti-tumor humoral immunity in established tumors, and is associated with improved T cell infiltration and response to anti-PD1 immunotherapy.

## Methods

### Tumor treatments and tumor measurements

Adult BALB/c mice (Envigo, Indiana, U.S.) at 6–8 weeks of age were inoculated with 1x10^5^ CT26 colon cancer cells into the left lower flank. Tumors were left to establish for 7 days post-injection, at which point they were palpable but not clearly measurable. Cages were randomly assigned to a treatment group. Clinical grade cobimetinib (GDC-0973, XL-518) was manufactured by Genentech, Inc. and acquired from an outpatient pharmacy. A 1.9mM cobimetinib stock solution was made by dissolving one 20 mg cobimetinib tablet in vehicle consisting of 20% DMSO and water. The MEKi group received 200ul of cobimetinib solution (approximately 7.5 mg/kg of cobimetinib) three times weekly via intraperitoneal injection, whereas the control group received vehicle only.

For tumor growth and depletion studies, the cobimetinib and control groups also received isotype antibodies. The PD1i group received vehicle solution plus 10 mg/kg anti-mouse PD-1 (Clone RMP1-14, Bio X Cell) three times per week. The combination group received both cobimetinib and anti-mouse PD-1. For depletion experiments, mice were additionally injected with 250 μg of anti-CD8 (YTS 169.4, Bio X Cell), anti-CD4 (Clone YTS 191, Bio X Cell), anti-CD19 (Clone 1D3, Bio X Cell) and appropriate isotype controls, for 3 days prior to initiation of cobimetinib treatment, and also on day 0, 24, and 27 of cobimetinib treatment. Tumor length and width were assessed three times weekly using caliper measurements, with the length assigned to the longest cross-sectional tumor diameter. Tumor volume was calculated as (tumor volume = (length*width^2^)/2. Tumor volume was assessed until tumors reached 20x20mm, at which point the mice were euthanized.

All animal studies were reviewed and approved by the Johns Hopkins Institutional Animal Care and Use Committee (ACUC) and Biohazards Committee. All efforts were made to limit animal pain and discomfort. Mice were monitored twice-daily by the Johns Hopkins Institutional Animal Care, and at least twice a week by the investigators to assess for animal suffering or distress such as but not limited to ruffled fur, weight loss, hunched posture, labored respiration, cyanosis, or signs of morbidity due to the growth of tumor or drug treatment. Feed and water were provided ad libitum, and mice were euthanized by CO2 narcosis.

### In Vitro B cell analysis

Splenocytes were harvested from adult BALB/c mice (Envigo, Indiana, U.S.). After the mice were euthanized, the spleen was resected under sterile conditions, resuspended in complete media, and filtered through a 40 μm cell strainer (Falcon, 352340). After spin down, red blood cells were lysed using Ammonium-Chloride-Potassium (ACK) lysing buffer (Avantor) and quenched in complete media. Cells were counted and resuspended at 1 x 10^8^ cells/mL in PBS containing 2% FBS and 1 mM EDTA. B cells were subsequently isolated using a B cell negative isolation kit (EasySep Cat 19854). The purity of B cells was confirmed to be >95% by FACs. Isolated B cells were counted and resuspended at 1*10^6^ cells/ml in complete media, and 500 ul (5*10^5^ cells) were plated in wells of a 48-well flat bottom plate. The isolated B cells were stimulated with goat anti-mouse IgM (1 mg/ml, catalog 1021–01, SouthernBiotech) and anti-CD40 (8 mg/ml, clone FGK4.5/ FGK45, Bio X Cell) or isotype control. 0.5 μM of cobimetinib or vehicle was subsequently added from a 1 mM stock solution to each well. The plate was incubated at 37 degrees C and 5% CO2 for 48 hours. Cells were washed and stained for FACs analysis.

### In Vivo B cell analysis

Adult BALB/c mice were inoculated with CT26 tumors in the left flank and subsequently treated with cobimetinib or vehicle started at day 7 as described above (see tumor treatments). After two weeks of cobimetinib treatment we harvested the left inguinal lymph node (tumor draining lymph node) as well as right inguinal lymph node (non-tumor draining lymph node). Nodes were resuspended in complete media, passed through a 40 μm cell strainer (Falcon, 352340), and washed for FACs analysis.

### Co-culture experiments

Tumor-draining inguinal lymph nodes were harvested from ten cobimetinib-treated and ten vehicle-treated mice, as described above. The lymph nodes were subsequently pooled together from each treatment group for B cell isolation. After washing, cells were counted and resuspended at 1 x 10^8^ cells/mL in PBS containing 2% FBS and 1 mM EDTA. B cells were subsequently isolated using a B cell negative isolation kit (EasySep Cat 19854). B cells were resuspended at 1*10^6^ cells/ml in complete media. CD8 T cells were subsequently isolated from three non-tumor bearing adult BALB/c mice spleens using a negative isolation kit (EasySep Cat 19853) and resuspended at 1*10^6^ cells/ml in complete media. B cells and T cells were subsequently co-cultured by combining 1*10^5^ of isolated T cells with 0, 1*10^5^, or 2*10^5^ of isolated B cells in 300 ul of complete media in a flat-bottom 96 well plate. For proliferation assays, the isolated T cells were first labelled with CFSE (Cell Trace Invitrogen) prior to co-culture. T cells were subsequently stimulated using anti-CD3- and anti-CD28-coated magnetic Dynabeads according to the manufacturer protocol (Gibco 11452D). Proliferation was assessed 48 hours later by FACs. For T cell intracellular staining assays, isolated T cells were co-cultured by combining 1*10^5^ of isolated T cells with 0, 1*10^5^, or 2*10^5^ of isolated B cells in 300 uL of complete media in a round-bottom 96 well plate along with goat anti-mouse IgM (1 mg/ml, catalog 1021–01, SouthernBiotech) to activate the cultured B cells. T cells were then stimulated using anti-CD3 and anti-CD28-coated magnetic Dynabeads as described above for 48 hours. Intracellular staining for interferon gamma and granzyme B expression was performed after permeabilizing cells using the Intracellular Fixation and Permeabilization Kit (88-8824-00, eBioscience) according to manufacturer protocol.

### Anti-Tumor IgG

To assess anti-tumor antibodies, adult BALB/c mice were inoculated with CT26 tumors in the left flank and subsequently treated with cobimetinib or vehicle started at day 7 as described above (see tumor treatments). At seven or 14 days after initiation of treatment with cobimetinib (21 days after tumor inoculation), a volume of <100 μl of blood was collected by tail bleed in a heparin tube. The collected blood was spun down at 15000 RPM for 10 minutes, and the supernatant was collected and stored at -80 degrees C. At a later time, 3*10^5^ cultured CT26 tumor cells were resuspended in 200 uL of FACs buffer containing serum from the non-treated mice at various dilutions. The samples were washed three times and a fluorochrome-conjugated goat anti-mouse IgG secondary antibody was applied (Thermo Fisher Scientific, 17-4010-82). A 1:200 dilution of serum to FACs buffer chosen for subsequent Anti-Tumor IgG experiments since 50% of tumor cells were stained positive using this dilution of serum (S2 Fig). 200 ul of FACs buffer and 1 uL of the serum from each mouse were added to each respective well as a primary antibody for 60 minutes at 4 degrees C. Mouse serum from a non-tumor bearing BALB/c mouse was used as a negative control. The samples were then stained with the fluorochrome-conjugated goat anti-mouse IgG secondary antibody as described above. The percentage of tumor cells staining positive for anti-tumor IgG was assessed by FACs.

### Quantitative immunohistochemistry

Adult BALB/c mice were inoculated with CT26 tumors in the left flank and subsequently treated with cobimetinib or vehicle started at day 7 as described above (see tumor treatments). After two weeks of treatment, mice were euthanized and their tumors were resected and placed in a 10% neutral buffered formalin fixative before paraffin embedding and sectioning at 5 μm. Immunolabeling for CD8 and CD4 was performed on formalin-fixed, paraffin embedded sections on a Ventana Discovery Ultra autostainer (Roche Diagnostics).

Briefly, following dewaxing and rehydration on board, epitope retrieval was performed using Ventana Ultra CC1 buffer (catalog# 6414575001, Roche Diagnostics) for 64 minutes at 96 degrees C (for CD8 staining) and for 84 minutes at 96 degrees C (for CD4 staining). Primary antibody, anti-CD8 (1:125 dilution; catalog# 14-0195-82, ThermoFisher Scientific), or anti-CD4 (1:250 dilution; catalog# 50134-R001, SinoBiological), were applied at 36 degrees C for 60 minutes. Primary antibodies were detected using an anti-rabbit HQ detection system (catalog# 7017936001 and 7017812001, Roche Diagnostics) followed by Chromomap DAB IHC detection kit (catalog # 5266645001, Roche Diagnostics), counterstaining with Mayer’s hematoxylin, dehydration, and mounting.

Whole slides were scanned at 20x objective equivalent (0.49 microns/pixel) using a digital slide scanner (Nanozoomer, Hamamatsu). Image analysis (HALO Indica Labs) was used to determine the density (# of cells/surface area analyzed) of CD8 or CD4 expressing lymphocytes within the tumor compartment.

### Flow cytometry and B cell suppression assay

Cells were washed with refrigerated PBS, stained with Live/Dead Fixable Aqua (Life Technologies) for 20 minutes on ice in the dark, washed with refrigerated PBS, and stained for surface markers diluted in FACS buffer (PBS with 1% FBS and 0.1% NaN3) for 30 minutes on ice in the dark. Cells were washed 3 times with FACS buffer, and either immediately on a CytoFLEX Flow Cytometer (Beckman Coulter). FACS analysis was performed using CytExpert Software (Beckman Coulter) and FlowJo 10.5.3 (FlowJo, LLC). An example of representative gating and analysis can be seen in S1 Fig.

### Tumor microenvironment gene expression analysis

To determine the effects of B cells in the tumor microenvironment, adult BALB/c mice were inoculated with CT26 tumors and treated with cobimetinib with or without B cell depleting antibody as described above. Once tumors grew to 20 mm x 20 mm, tumors were excised and further dissociated into single-cell suspensions using the Miltenyi Tumor Dissociation Kit (#130-096-730) and a GentleMACS Octo dissociator. RNA was extracted from dissociated tumors using the Direct-zol RNA kit (catalog# R2071, Zymo Research). For gene expression analysis, 100ng total RNA was used for hybridization per reaction using the PanCancer IO 360^™^ gene expression panel (NanoString Technologies). Data analysis was performed using nSolver 4.0 (NanoString Technologies).

### Statistical analysis

Data were analyzed using Prism 4.0 (GraphPad Software, Inc.). We used Student’s t-test for assessing statistics involving two experimental groups or conditions, and one-way ANOVA with correction for multiple comparisons by post hoc Tukey’s test for assessing statistics involving more than two experimental groups. Differences were considered significant when the P-value was <0.05. For gene expression data, statistically differentially expressed genes were determined using the DE call function in nSolver 4.0.

## Results

### MEK inhibition reprograms B cells in Vitro in the setting of BCR engagement

**Fig 1:**
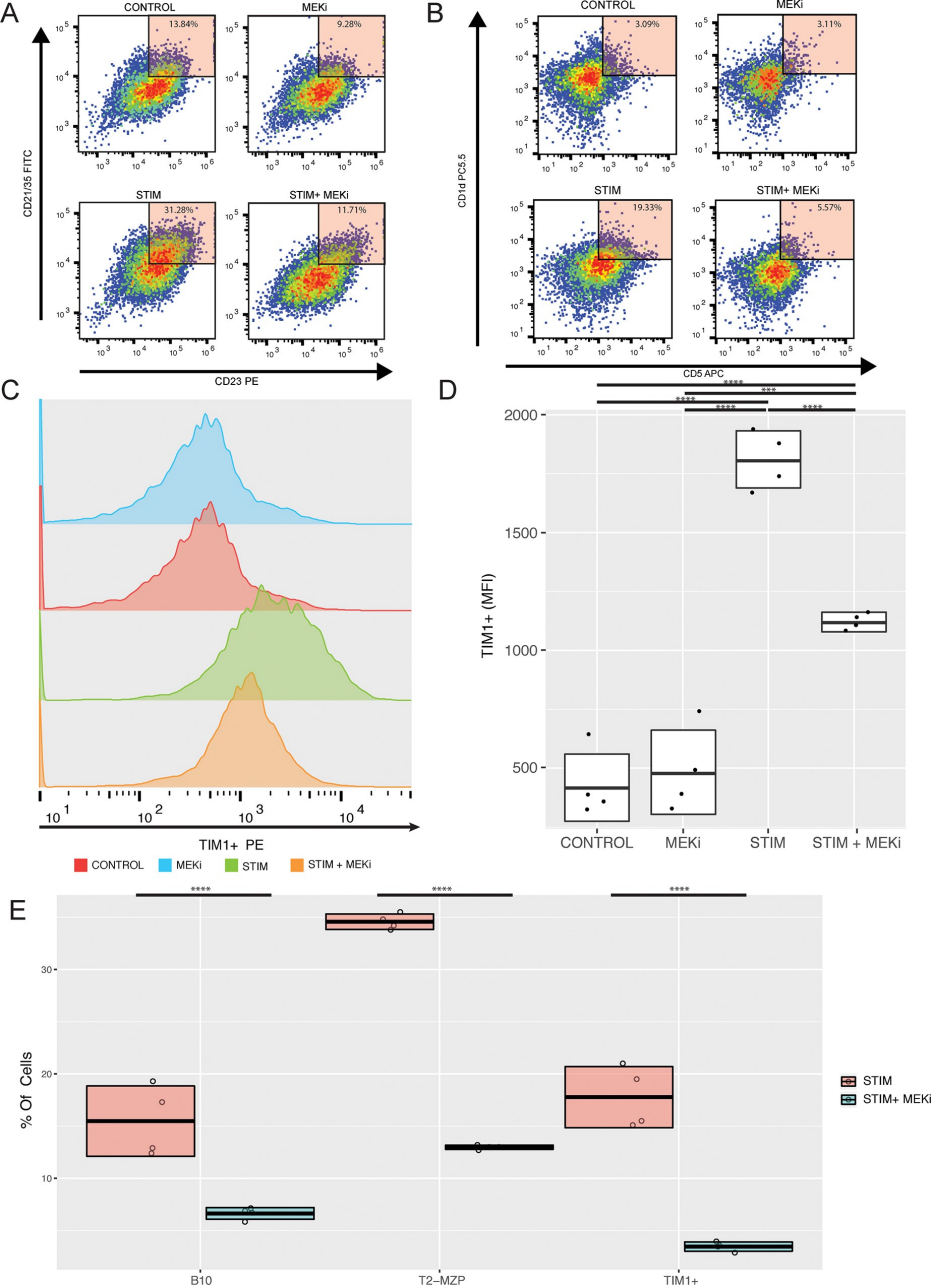
Cobimetinib (MEKi) inhibits T2-MZP Bregs, B10 Bregs, and TIM1+ Bregs in the setting of BCR engagement in vitro. (A) Representative flow plots of isolated splenic B cells cultured for 48 hours, with and without stimulation (anti-IgM and anti-CD40), and with and without cobimetinib. The percentage of T2-MZP cells (CD21hi, CD23hi, CD24hi) was modestly decreased with cobimetinib in unstimulated B cells (top). The percentage of T2-MZP cells was greatly increased with stimulation (top left vs. bottom left), and this increase was largely attenuated by the addition of cobimetinib to the culture media (bottom right). (B) The proportion of B10 cells (CD5+CD1dhi) similarly increased with stimulation, and this increase was largely attenuated with cobimetinib. These observed changes in B10 cell subsets were driven by changes in CD5 expression rather than CD1d expression. (C) The proportion of TIM1+ B cells was increased with stimulation, and this effect was attenuated by cobimetinib. (D) The proportion of TIM1+ B cells was increased with stimulation, and this effect was attenuated by cobimetinib represented as MFI values. One way ANOVA followed by a post Tukey’s test was used to determine statistical significance between multiple groups. (E) Summary of the percentage of Breg subsets from isolated B cells cultured with anti-IgM and anti-CD40, with or without cobimetinib. Student’s t-test was used to determine statistical significance. (Total number of events collected per sample = 30,000).

To identify a potential relationship between MAPK pathway inhibition and B cell mediated immune responses, we first assessed the effect of the highly specific MEK inhibitor GDC-0973 (cobimetinib)[15] on isolated splenic B cells in vitro. When B cells isolated from a BALB/c mouse using a negative isolation system were cultured in vitro for 48 hours, only a small portion of these unstimulated isolated B cells appeared to express Breg markers, and the portion of Bregs was also mostly unchanged in the presence of cobimetinib except for a small decrease in the number of T2-MZP cells (CD21^hi^, CD23^hi^, and CD24^hi^) (Fig 1). Therefore, at doses that exceeded physiologic relevance, MEK inhibition appeared to have only minor effects on unstimulated B cell function in vitro.

Because the MAPK pathway partially mediates B cell responses to antigen stimulation [14], we further investigated the effects of MEK inhibition in the setting of BCR engagement. Using anti-IgM antibodies to stimulate BCR signaling, and in combination with anti-CD40 co-stimulation to further enhance the number of Bregs, we observed marked phenotypic B cell changes in the presence of cobimetinib (Fig 1). Whereas anti-IgM and anti-CD40 stimulation increased the percentage of B cells that expressed Breg markers CD5 and CD1d (B10 cells), TIM1 (TIM1+ B cells), and CD21, CD23, and CD24 (T2-MZP cells) these effects were partially abrogated by MEKi. In particular, expression of CD5 and TIM1 were significantly decreased in the setting of cobimetinib, whereas the expression of CD1d and CD24 were less affected by cobimetinib. While MEK inhibition with cobimetinib had marked effects on the expression of Breg markers, the percentage of live B cells was not significantly affected by cobimetinib. Together these observations suggested that cobimetinib can inhibit B cell function in the setting of BCR engagement in vitro, by reducing the development of Bregs.

### Effects of MEK inhibition on B cells in Vivo

We next asked whether the observed effects of MEK inhibition on Bregs are also observed in vivo. BALB/c mice were inoculated with CT26 cells, a colon carcinoma cell line that is known to be homozygously mutated at KRAS G12D and sensitive to MEK inhibitor therapy [16]. After 7 days, when tumors became established, we treated the mice three times weekly with cobimetinib or vehicle. Consistent with prior reports, tumor growth was delayed in the cobimetinib group versus the vehicle group, but no tumors regressed or were fully eliminated. At 21 days from the time of tumor inoculation, we euthanized the mice and characterized the B cells in the tumor draining lymph nodes and corresponding non-tumor draining lymph nodes. We focused our analysis on the tumor draining lymph nodes as this is where tumor-associated Bregs are known to accumulate and facilitate tumor progression [13].

**Fig 2:**
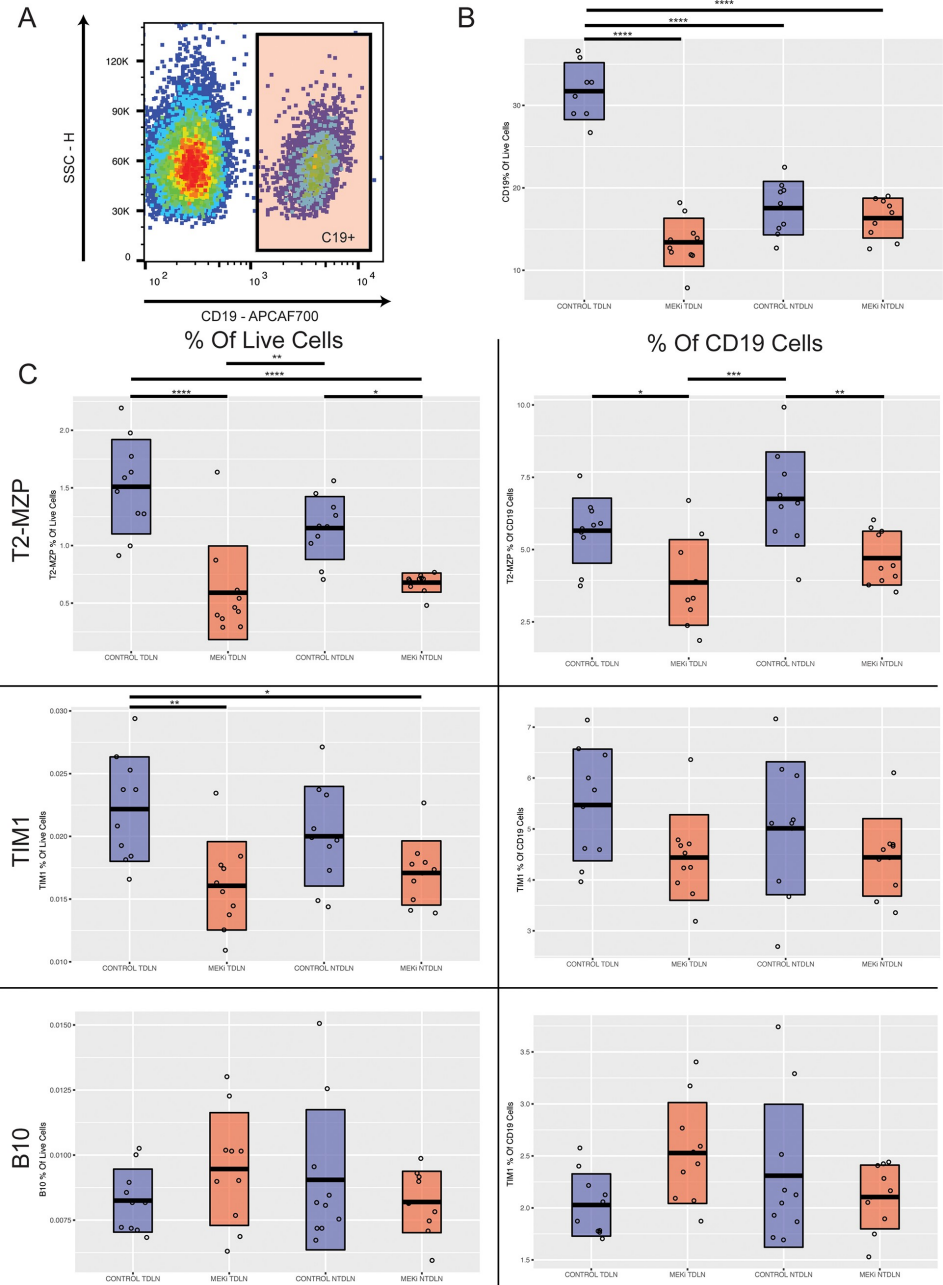
MEK inhibits Bregs in vivo. (A) Representative plot for CD19+ population of total live lymphocytes in tumor draining lymph node (B) The total percentage of B cells (CD19+ cells as a percentage of total live lymphocytes) in tumor and non-tumor draining lymph nodes of mice treated with cobimetinib or vehicle. (C) Percentage of T2-MZP cells (CD19+, CD21^hi^, CD23^hi^, CD24^hi^), Tim1+ B cells, and B10 cells (CD19+, CD5+, CD1d^hi^) in tumor and non-tumor draining lymph nodes of mice treated with cobimetinib or vehicle. One way ANOVA followed by a post Tukey’s test was used to determine statistical significance between groups. (n = 10, total number of events collected per sample = 30,000).

There was a significant increase in the total percentage of B cells in the tumor draining lymph nodes of control treated mice when compared with the non-tumor draining nodes of these mice (Fig 2A). By contrast, the percentage of B cells in the tumor draining nodes of the cobimetinib-treated mice was lower than the non-tumor draining nodes of these mice. Therefore, the percentage of B cells in tumor draining lymph nodes of control mice was more than double those of cobimetinib-treated mice (28.19% vs. 12.9%, p <0.0001).

We examined the phenotypes of the B cells in the tumor draining and non-tumor draining nodes of vehicle and cobimetinib treated tumor bearing mice. The percentage of T2-MZP Bregs were reduced in the tumor draining nodes of cobimetinib treated mice versus the mice that received vehicle (Fig 2B). As both the B cell fraction, and the percentage of these B cells with T2-MZP differentiation were reduced in tumor draining nodes of the cobimetinib treated mice, the result was a marked decrease in the percentage of T2-MZP as a function of total lymphocytes in the tumor draining nodes of cobimetinib-treated mice as compared to vehicle (Fig 2C). The total number of lymphocytes per lymph node were similar between cobimetinib and vehicle treated mice, and therefore, this difference in T2-MZP percentage represented a decrease in absolute T2-MZP cells. Although the total percentage of B cells in the non-tumor draining nodes was similar between cobimetinib and vehicle treated mice, a lower percentage of the B cells in the cobimetinib arm had T2-MZP differentiation. Therefore, cobimetinib treated mice had a lower number of T2-MZP Bregs in both tumor draining and non-tumor draining nodes, with the lowest fraction of T2-MZP Bregs in the cobimetinib tumor draining nodes and the highest in vehicle tumor draining nodes (Fig 2C).

Tim1+ Bregs were less frequent than T2-MZP Bregs in tumor and non-tumor draining lymph nodes. However, as with T2-MZP Bregs, the number of Tim1+ Bregs was significantly reduced in the tumor draining nodes of cobimetinib-treated mice as compared to vehicle treated mice. B10 Bregs have previously been described to be restricted to the spleen and were infrequently observed in tumor and non-tumor draining lymph nodes in our model. The total percentage of B10 cells were similar across all the groups and were observed at very low percentages of total events.

**Fig 3:**
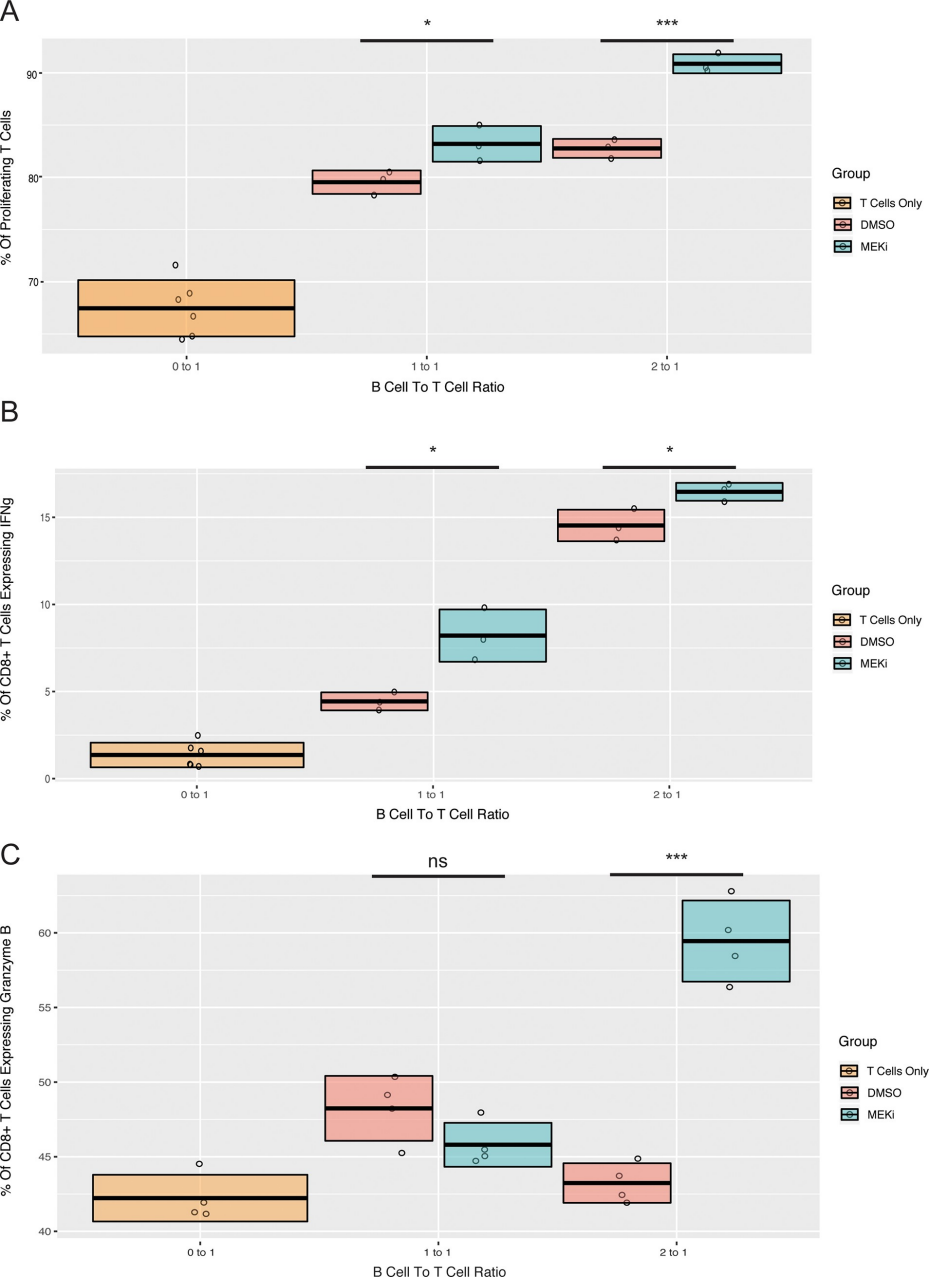
T cells isolated from three Balb/C mice were cocultured with pooled B cells isolated from the tumor draining nodes of cobimetinib and vehicle treated mice, at a ratio of 1 B cell to every T cell and 2 B cells to every T cell. (A) At 48 hours more proliferation of T cells was observed in the coculture with B cells from cobimetinib-treated mice than vehicle mice. (B) At 48 hours more INFg release by T cells was observed in the coculture with B cells from cobimetinib-treated mice than vehicle mice. (C) At 48 hours more Granzyme B release by T cells was observed in the coculture with B cells from cobimetinib-treated mice than vehicle mice. A student’s t-test was used to determine statistical significance between groups at the same ratio of B cells to T cells. (Total number of events collected per sample = 30,000).

To confirm a functional difference in the phenotype of B cells from the tumor draining nodes of cobimetininb and vehicle treated mice, we co-cultured at activated CD8 T cells with pooled B cells isolated from tumor draining lymph nodes of mice treated with cobimetinib or vehicle. The CD8 T cells were labeled before co-culture with carboxyfluorescein succinimidyl ester (CFSE) and were stimulated in the presence of the tumor-associated B cells with anti-CD3- and anti-CD28-coated magnetic dynabeads for 48 hours. There was a significant difference in the percent of T cells proliferating, with more proliferation of the T cells co-cultured with tumor draining B cells from the cobimetinib treated mice (Fig 3A). However, the presence of B cells from both cobimetinib and vehicle mice increased proliferation relative to the control group of CD8 T cells cultured without B cells. These results are consistent with the hypothesis that heterogeneous populations of B cell from both groups contain B cells that support T cell expansion, but that the B cells obtained from the cobimetinib treated mice contain fewer suppressive B cells than the vehicle treated mice. At higher co-culture ratios of B to T cells, the T cells cultured with B cells derived from tumor draining lymph nodes of cobimetinib treated mice also exhibited more interferon-gamma and granzyme B expression than those which received vehicle alone (Fig 3B and 3C).

### B cells augment anti-tumor immunity in the setting of MEK inhibition

In addition to their role in promoting immune tolerance, B cells can also promote anti-tumor immunity through the secretion of antigen-specific antibodies and by facilitating T-cell-mediated immune responses that inhibit tumor development [17]. Since MEK inhibition inhibits Bregs, we sought to investigate whether B cell anti-tumor immunity is preserved in the setting of MEK inhibition, or whether MEK blocking agents inhibit both anti-tumor and pro-tumor B cell activity.

**Fig 4:**
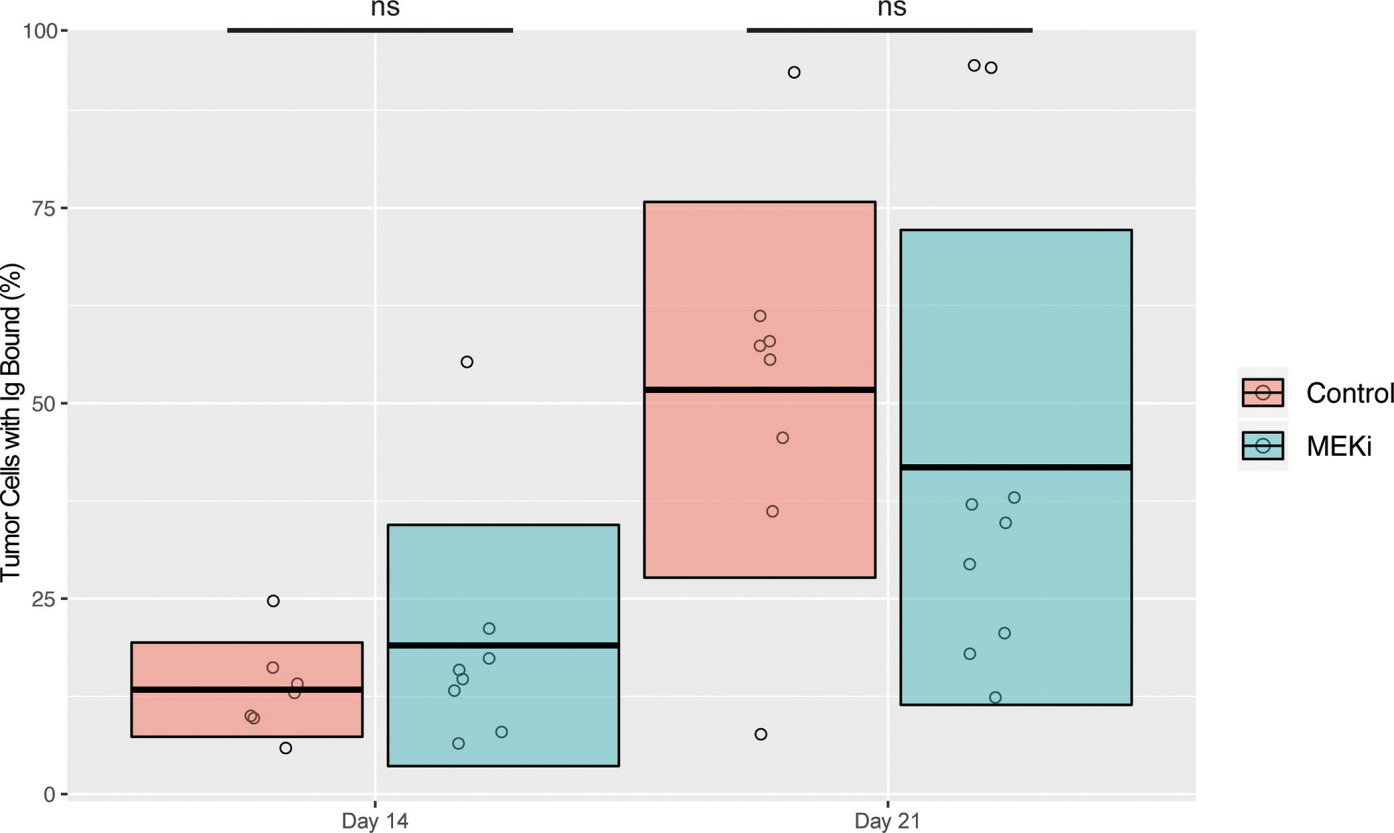
Adult BALB/c mice were inoculated with CT26 tumors and treated with cobimetinib or vehicle beginning at day 7. At day 14 or 21 after tumor inoculation, titers of anti-CT26 antibodies were similar in cobimetinib or vehicle treated mice. A student’s t-test was used to determine statistical significance between groups at the same time point. (Total number of events collected per sample = 30,000).

To assess the effects of MEK inhibition on anti-tumor humoral immunity, we tested anti-tumor antibody titers in tumor bearing mice treated with or without cobimetinib. BALB/c mice inoculated CT26 tumors in the left flank and subsequently treated with cobimetinib or vehicle beginning at day 7 after tumor implantation. Serum was collected from each mouse at 14 days and 21 days after tumor inoculation. We subsequently quantified anti-CT26 antibody titers from each mouse by suspending CT26 tumor cells in the presence of the collected serum and assessing the percentage of CT26 tumor cells staining positive for anti-tumor IgG by FACs. There was no significant difference in the percent of CT26 tumor cells with IgG bound (Fig 4) at either 14 or 21 days, indicating that MEK inhibition did not inhibit anti-tumor humoral immunity.

**Fig 5:**
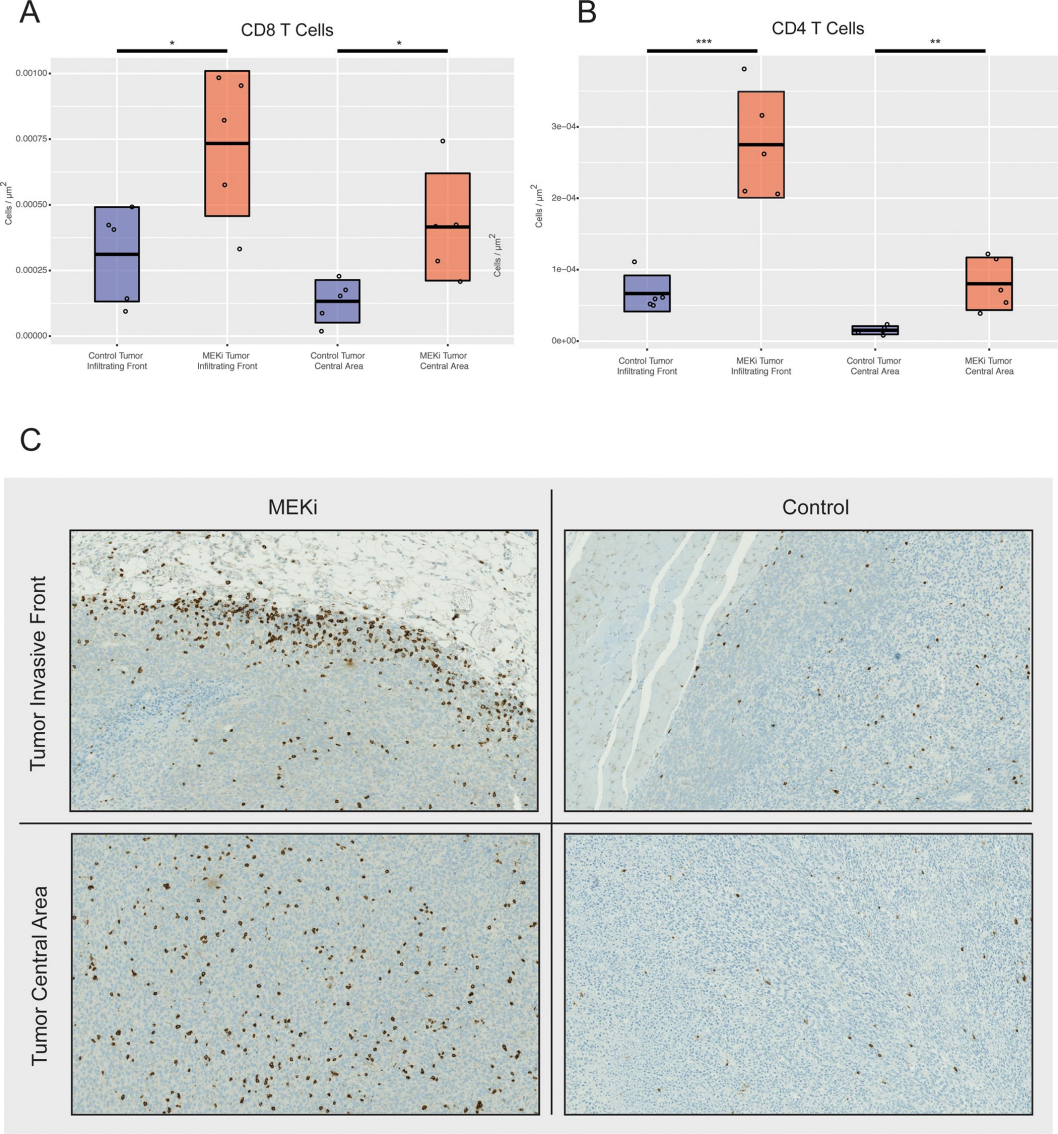
CT26 tumors were treated with cobimetinib or vehicle and then the density of CD8 and CD4 T cells was measured by quantitative immunohistochemistry. Cobimetinib treatment resulted in a modest increase in CD8 T cell density and a marked increase in CD4 T cell density in both the tumor invasive front and central areas. A student’s t-test was used to determine statistical significance between groups at the tumor invasive front and in the tumor central area. (Total number of events collected per sample = 30,000).

Since B cells also facilitate T cell-mediated immune responses, we sought to confirm prior reports indicating that MEK inhibition induces the accumulation of T cells in established tumors [9]. B cells are particularly important for CD4 T cell priming, and Bregs are important for suppressing CD4 T cell responses, so we hypothesized that MEK inhibition would specifically augment the number of tumor infiltrating CD4 T cells. To test this hypothesis, we again treated established CT26 tumors with cobimetinib or vehicle and then analyzed the density of CD8 and CD4 T cells by quantitative immunohistochemistry. Consistent with prior results, we found that the density of CD8 T cells was modestly increased in MEK treated tumors as compared to vehicle. However, the density of CD4 T cells was increased more significantly than CD8 T cells, approximately three-fold as compared to vehicle treated tumors (Fig 5).

**Fig 6:**
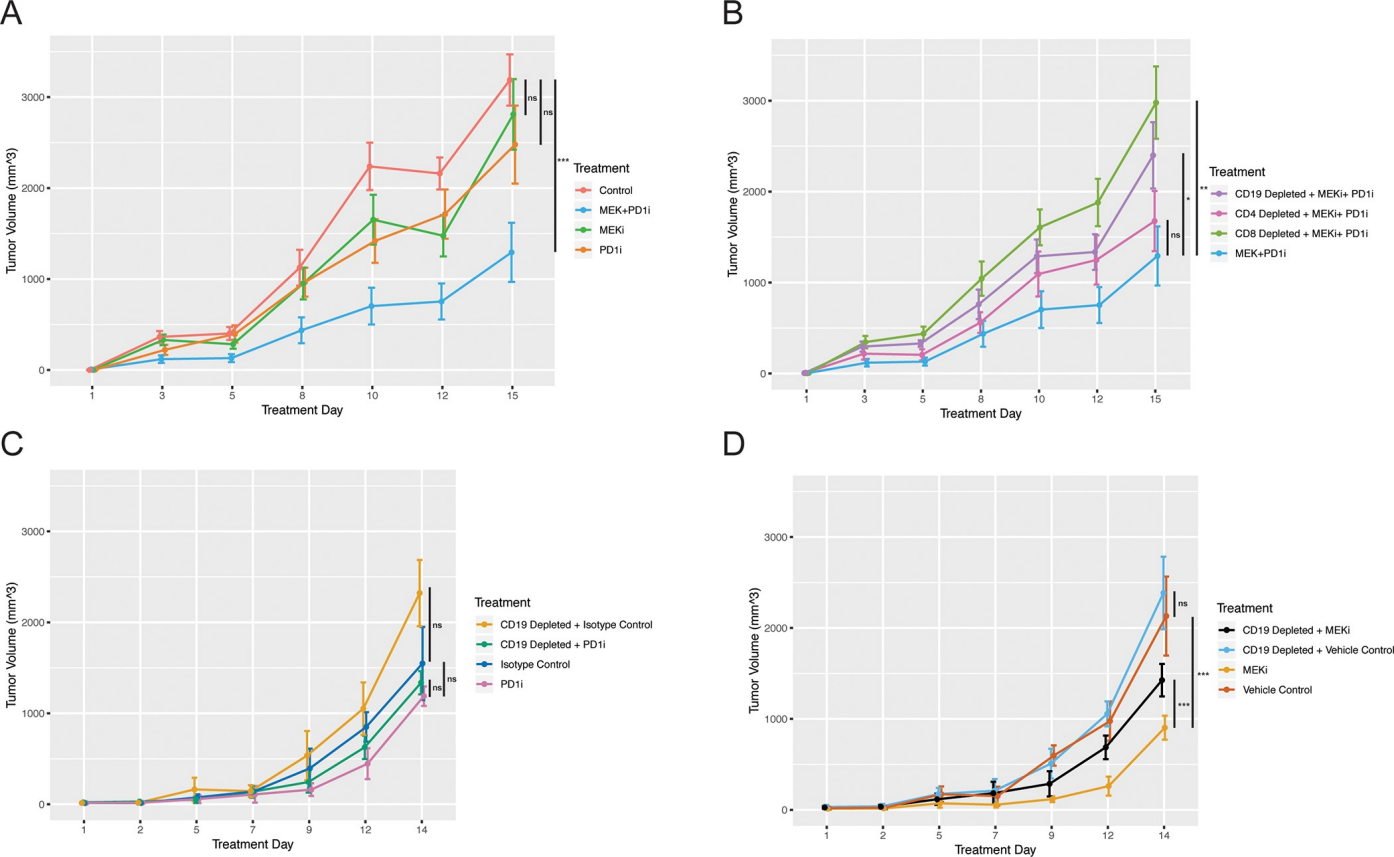
Treatment with the MEK inhibitor cobimetinib (MEKi) and anti-PD-1 therapy (PD1i) did not significantly delay tumor progression in the CT26 tumor model, whereas combination therapy markedly delayed tumor growth. Depletion of CD4 T cells, CD8 T cells, and CD19 B cells with the injection of anti-CD8, anti-CD4, and anti-CD19 antibodies prior to and at the time of MEK inhibition plus anti-PD1 treatment identified that the effect of MEKi plus PD1i was most dependent on CD8 T cells. However, depletion of CD19+ cells also resulted in significantly faster tumor growth than MEKi plus PD1i. Depletion of CD4 positive T cells had no significant effect on the rate of tumor progression. Depletion of CD19+ cells in MEK inhibitor treated tumors induced faster tumor growth. A student’s t-test was used to determine significance at the last time point. (n = 5).

In preclinical studies, MEK inhibitors have shown significant anti-tumor activity in preclinical models when combined with PD-1 immunotherapy in a CT26 tumor model, but whether B cells meaningfully contribute to the anti-tumor activity of MEK inhibition plus anti-PD1 immunotherapy is not known [8][9]. To understand the relative contributions of each cell subset in the setting of MEK inhibitor plus PD-1 inhibitor, we inoculated BALB/c mice with CT26 cells, then depleted CD8+ T cells, CD4+ T cells, and B cells, by injecting anti-CD8, anti-CD4, and anti-CD19 antibodies prior to and at the time of MEK inhibition plus anti-PD1 treatment. Consistent with prior reports [9], MEK inhibition and PD1 inhibitor treatment each had modest single agent activity in the CT26 model whereas combination therapy markedly delayed tumor growth. Although tumor growth was significantly delayed in the combination group, we did not observe tumor regressions in any mice. Depletion of immune lineages revealed that the activity of MEK inhibitor plus PD1 inhibitor was most dependent on the activity of CD8 T cells, but depletion of either CD4 T cells or B cells also attenuated the clinical activity of the combination therapy. The anti-tumor activity of B cells in the setting of MEK inhibitor plus PD1 inhibitor was more than the anti-tumor activity of CD4 T cells, but less than the activity of CD8 T cells (Fig 6B). B cell depletion, in the context of both MEK inhibitor plus PD1 inhibitor therapy (Fig 6B) and MEK inhibitor monotherapy (Fig 6D), resulted in faster tumor growth, indicating that B cells play a significant role in anti-tumor immunity in the context of MEK inhibition. In contrast, B cell depletion did not significantly affect tumor growth in mice treated with vehicle or PD1 inhibitor monotherapy (Fig 6C).

**Fig 7:**
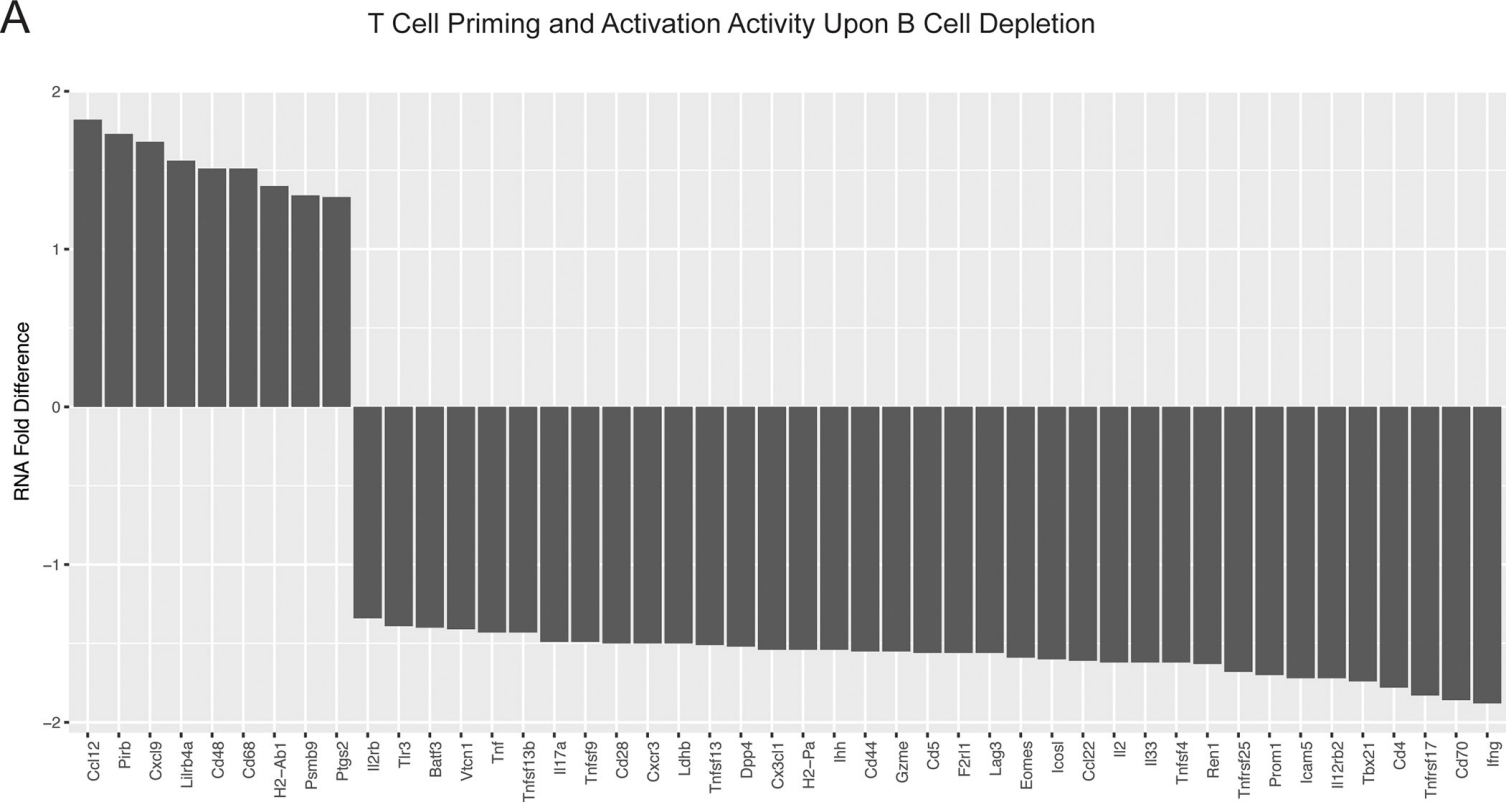
Gene expression analysis shows the effects of B cell depletion on the tumor microenvironment in the context of MEK inhibition. Three mice each were treated with MEK inhibition with and without B cell depletion, and RNA level fold changes are plotted for genes associated with T cell functional activity according to NanoString Technology’s probe annotations. All statistically significantly differentially expressed genes within the T cell functional activity panel, as determined by nSolver’s DE Call function, are shown.

To further elucidate the effects of B cells on anti-tumor immunity in the context of MEK inhibition, we explored the effects of B cell depletion on gene expression profiling within the tumor microenvironment in MEK treated mice. Within the MEK treated mice, B cell depletion with an anti-CD19 antibody resulted in reduced expression of multiple genes involved in T cell priming and activation, such as interferon-gamma and granzyme B expression, and T cell response to antigen encounter, such as CD44 expression (Fig 7). Together these findings indicate that B cells in the setting of MEK inhibition may have an immunomodulatory role on the anti-tumor T cell response.

## Discussion

In summary, we show that treatment with a MEK inhibitor reduces Bregs *in vitro* and *in vivo* in a model of colorectal cancer. The mechanism through which MEK inhibition results in decreased Bregs is unclear, but may be related to disruption of chronic BCR signaling, resulting in decreased upregulation of specific suppressive surface molecules. However, our data suggests that MEK inhibitor therapy does not impede anti-tumor humoral immunity, and suggests a positive role for B cells in the anti-tumor immune response in the context of MEK inhibition. In support of this idea, B cell depletion in the context of MEK inhibitor therapy resulted in reduced expression of multiple genes involved in T cell priming and activation and increased tumor growth kinetics. The MAPK pathway, for which MEK is a critical downstream intermediary, is a key pathway involved in both tumor cell survival and lymphocyte response to antigen stimulation [9]. Therefore, pharmacological inhibition of MEK can have effects on both tumor cells and immune cells that together may modulate tumor immunity, particularly when MEK inhibitions are combined with systemic immunotherapies such as inhibitors of the PD-1/PD-L1 axis.

Our results showing a decrease in Bregs with MEK inhibition contrasts with prior observations indicating that MEK inhibitors may be immunosuppressive in some contexts. For example, MEK inhibitors can profoundly inhibit T cell priming and proliferation and have been studied as a potential treatment for autoimmune disease [9,18–21]. We began our in vivo treatment with MEK inhibitors at seven days post tumor implantation when tumors were already established, at which point a critical level of lymphocyte priming may have already occurred. It is possible that earlier introduction of MEK inhibitor treatment, or a longer duration of treatment, may have produced different results due to early inhibition of lymphocyte priming. However, the treatment paradigm used in this study likely reflects the use of MEK inhibitors in humans with advanced metastatic cancer, where significant presentation of antigens would have already occurred prior to treatment initiation. Our observation that the inhibition of MEK can augment rather than abolish anti-tumor B cell immunity in a preclinical tumor, and the evidence for an immunosuppressive effect of MEK inhibition in autoimmune models, suggests that MEK inhibition may have context-dependent effects.

Targeted therapies are increasingly used in combination or sequentially with systemic immunotherapies, and therefore, it is important to understand the effects of specific targeted therapies including MEK inhibitors on both tumors and individual immune subsets. Although the combination of a MEK inhibitor and a PD-L1 inhibitor recently failed to show compelling efficacy in a pivotal clinical trial of colorectal cancer (IMblaze370), a tumor type for which MEK inhibition and immune checkpoint therapy have no meaningful activity in unselected patients, MEK inhibitors continue to be studied as a means of sensitizing tumors to immune checkpoint therapy in multiple other tumor types, particularly those in which immunotherapy has some single agent activity [22]. For example, MEK inhibitors and inhibitors of the PD-L1/PD1 axis are often used sequentially in the treatment of BRAF V600E mutated melanoma and are under investigation in combination [5,8]. Our findings provide novel insights into the immunomodulatory effects of MEK inhibitors, and suggest that disruption of chronic BCR signaling through MEK inhibition may inhibit Bregs. Our results support further investigation of MEK inhibitors in combination with systemic immunotherapies in tumors for which Bregs may be important for maintaining immune tolerance.

## Supporting information

S1 Fig Representative gating strategy wherein lymphocytes were gated for live cells followed by gating for single cells and then CD19+ subpopulations. Of the CD19+ group, the percentage of CD24+ CD21+ CD23+ cells were determined.
(TIF)

S2 Fig Representative CFSE proliferation assay histograms. **T Cells Only (Red) represents CD8+ T cells stimulated in the absence of B cells.** DMSO 2:1 (Blue) represents CD8+ T cells stimulated in the presence of 2 B cells for every T cell, where B cells were taken from DMSO treated mice. MEKi 2:1 (Orange) represents CD8+ T cells stimulated in the presence of 2 B cells for every T cell, where B cells were taken from MEK inhibitor treated mice.
(TIF)

S3 Fig Serum collected from five adult BALB/c mice 21 days after inoculated with CT26 tumors were serially diluted in FACs buffer to determine the optimal primary antibody dilution for mouse anti-tumor IgG experiments. 3*10^5^ cultured CT26 tumor cells were resuspended in the serum dilution, washed, and then stained with a fluorochrome-conjugated goat anti-mouse IgG secondary antibody. Mouse serum from a non-tumor bearing BALB/c mouse was used as a negative gating control. A 1:200 dilution of serum to FACs buffer chosen for subsequent anti-tumor IgG experiments because 50% of tumor cells were stained positive using this dilution of serum.
(TIF)
