# Supplementary figures and images for: MEK inhibition suppresses B regulatory cells and augments anti-tumor immunity
Source: PLoS One. 2019 Oct 31;14(10):e0224600. doi: 10.1371/journal.pone.0224600 (PMC6822709; doi:10.1371/journal.pone.0224600)

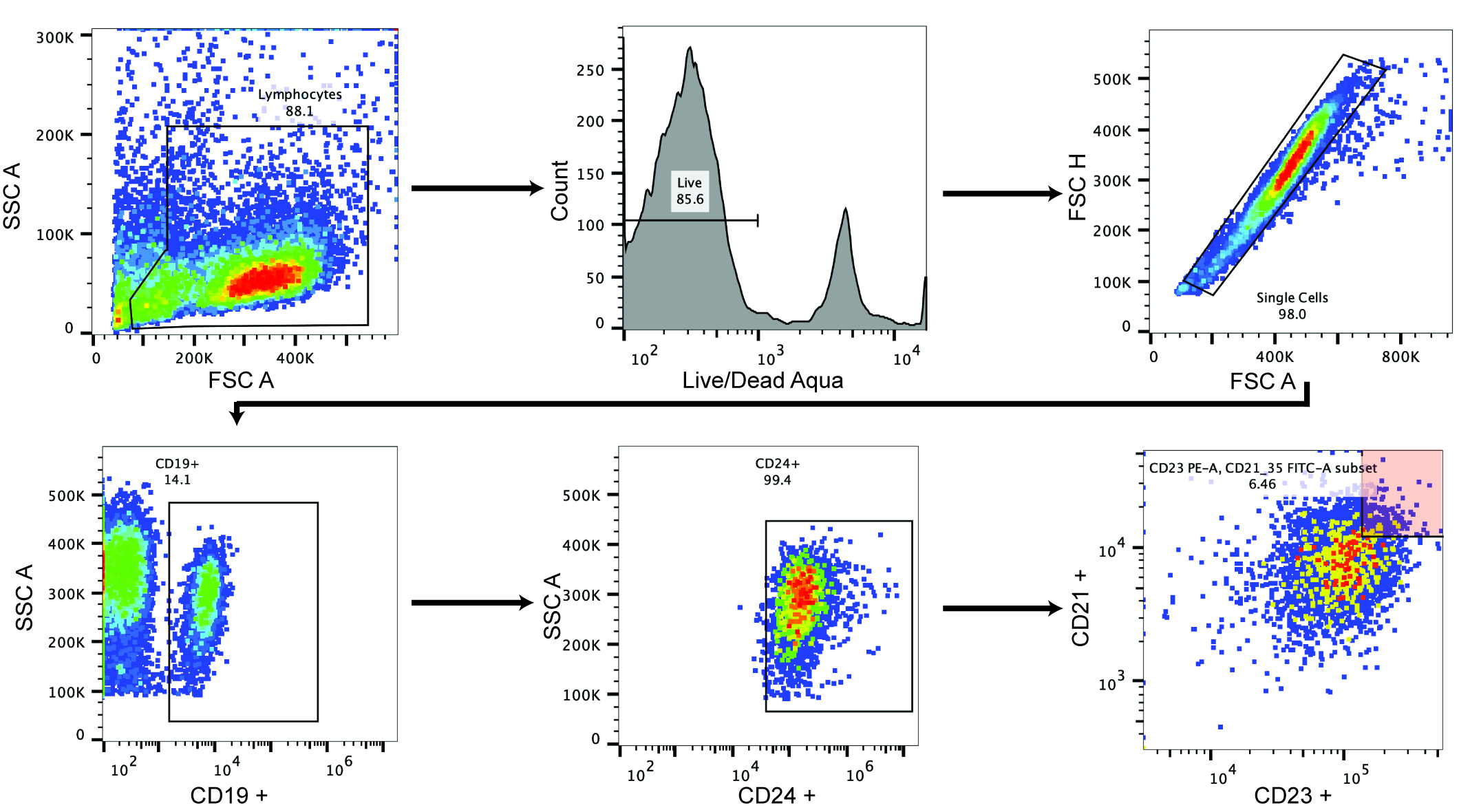

Supplement: S1 Fig — Of the CD19+ group, the percentage of CD24+ CD21+ CD23+ cells were determined. (TIF) [file pone.0224600.s001.tif]

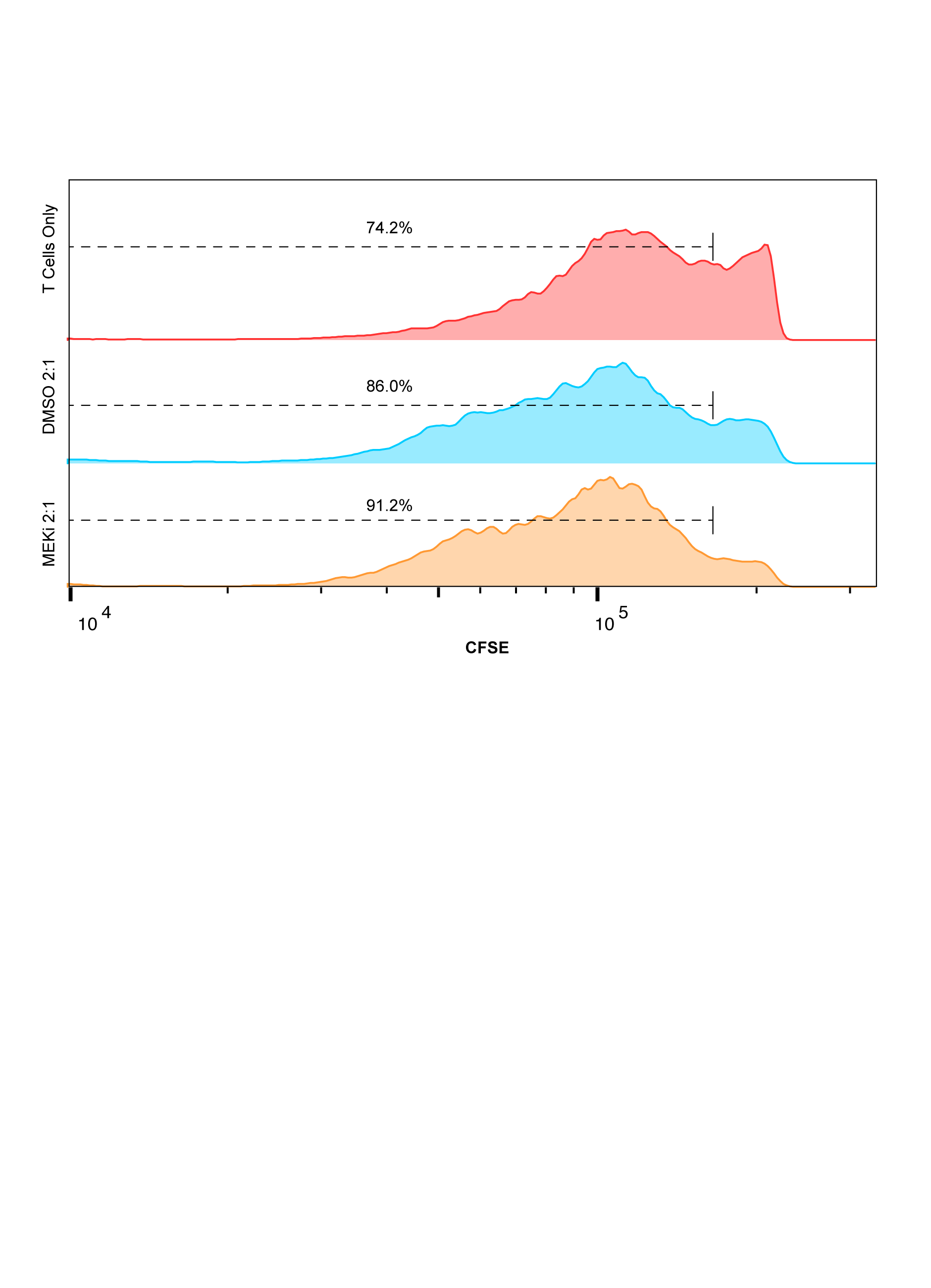

Supplement: S2 Fig — T Cells Only (Red) represents CD8+ T cells stimulated in the absence of B cells. DMSO 2:1 (Blue) represents CD8+ T cells stimulated in the presence of 2 B cells for every T cell, where B cells were taken from DMSO treated mice. MEKi 2:1 (Orange) represents CD8+ T cells stimulated in the presence of 2 B cells for every T cell, where B cells were taken from MEK inhibitor treated mice. (TIF) [file pone.0224600.s002.tif]

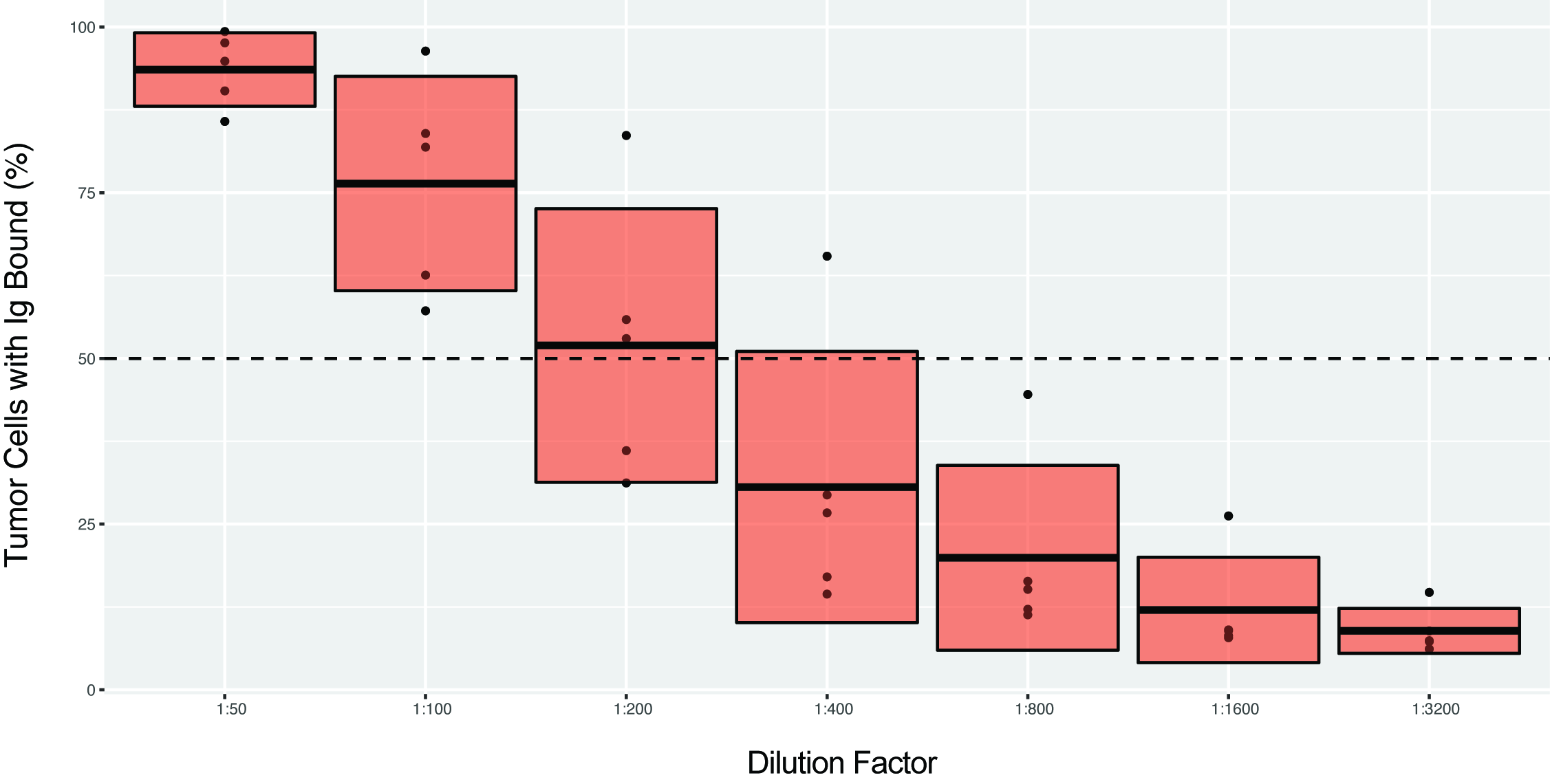

Supplement: S3 Fig — 3*105 cultured CT26 tumor cells were resuspended in the serum dilution, washed, and then stained with a fluorochrome-conjugated goat anti-mouse IgG secondary antibody. Mouse serum from a non-tumor bearing BALB/c mouse was used as a negative gating control. A 1:200 dilution of serum to FACs buffer chosen for subsequent anti-tumor IgG experiments because 50% of tumor cells were stained positive using this dilution of serum. (TIF) [file pone.0224600.s003.tif]
